# Functional Activation of Newborn Neurons Following Alcohol-Induced Reactive Neurogenesis

**DOI:** 10.3390/brainsci11040499

**Published:** 2021-04-15

**Authors:** Natalie N. Nawarawong, Chelsea G. Nickell, Deann M. Hopkins, James R. Pauly, Kimberly Nixon

**Affiliations:** 1 College of Pharmacy, The University of Texas at Austin, Austin, TX 78712, USA; natalie.nawarawong@austin.utexas.edu; 2 Department of Pharmaceutical Sciences, University of Kentucky, Lexington, KY 40536, USA; Chelsea.nickell@uky.edu (C.G.N.); dmhopk1@uky.edu (D.M.H.); jim.pauly@uky.edu (J.R.P.)

**Keywords:** adult neurogenesis, alcohol, c-Fos, ethanol, hippocampus, neural stem cell, neuronal ensemble, recovery, spatial learning

## Abstract

Abstinence after alcohol dependence leads to structural and functional recovery in many regions of the brain, especially the hippocampus. Significant increases in neural stem cell (NSC) proliferation and subsequent “reactive neurogenesis” coincides with structural recovery in hippocampal dentate gyrus (DG). However, whether these reactively born neurons are integrated appropriately into neural circuits remains unknown. Therefore, adult male rats were exposed to a binge model of alcohol dependence. On day 7 of abstinence, the peak of reactive NSC proliferation, rats were injected with bromodeoxyuridine (BrdU) to label dividing cells. After six weeks, rats underwent Morris Water Maze (MWM) training then were sacrificed ninety minutes after the final training session. Using fluorescent immunohistochemistry for c-Fos (neuronal activation), BrdU, and Neuronal Nuclei (NeuN), we investigated whether neurons born during reactive neurogenesis were incorporated into a newly learned MWM neuronal ensemble. Prior alcohol exposure increased the number of BrdU+ cells and newborn neurons (BrdU+/NeuN+ cells) in the DG versus controls. However, prior ethanol exposure had no significant impact on MWM-induced c-Fos expression. Despite increased BrdU+ neurons, no difference in the number of activated newborn neurons (BrdU+/c-Fos+/NeuN+) was observed. These data suggest that neurons born during alcohol-induced reactive neurogenesis are functionally integrated into hippocampal circuitry.

## 1. Introduction

Alcohol misuse remains a concerning public health crisis as a leading cause of preventable death due to the harmful impact it has on individuals and society [1,2,3]. In 2019 in the U.S., over 139 million people aged 12 and older reported alcohol use in the past month with 47.1% identifying as binge drinkers (≥5 drinks in males and ≥4 drinks in females over a 2 h period; [4] and 11.5% reporting heavy or excessive alcohol use [5]. Approximately 13% of U.S. adults meet Diagnostic and Statistical Manual V criteria of an alcohol use disorder (AUD) [6].

While excessive alcohol use impacts a wide array of organ systems within the body, brain damage by alcohol can lead to permanent cognitive impairments [7,8,9]. Imaging studies and postmortem examinations of the human brain have documented a loss of volume and mass with excessive alcohol drinking [10,11,12,13]. Furthermore, a binge-like pattern of drinking is associated with a greater likelihood of neurodegeneration [14,15,16,17]. While brain-wide volume loss has been identified, some areas of the brain are more vulnerable to the effects of alcohol. The hippocampus, an area of the brain important for learning and memory, is known to be more sensitive to the damaging effects of alcohol [18,19,20,21,22,23], for review see [24]. Alcohol exposure models have shown consistently that alcohol is toxic to hippocampal neurons, including dentate gyrus granule cells [16,18,25,26,27,28,29,30]. Accordingly, excessive alcohol consumption is associated with impaired hippocampal-based learning and memory in humans [13,31,32,33] as well as in animal models [30,34]. However, the brain, especially the hippocampus, recovers with abstinence, though the mechanism of this recovery is not known [35,36,37,38,39,40,41,42].

One means by which the hippocampus may recover is through adult neurogenesis, the ongoing generation of new neurons from neural stem cells (NSC; [43,44,45,46]; for review see [47,48]). Adult neurogenesis is well-accepted to occur within two regions of the brain, the subventricular zone of the lateral ventricles [49,50] and the subgranular zone (SGZ) of the hippocampus [43,51,52]. This study focuses on adult hippocampal neurogenesis due to hippocampal pathology in AUDs and that adult neurogenesis has been observed in the hippocampus of all mammals including humans, a debate that was recently resolved [53,54,55]. Newborn granule cells are derived from a population of NSCs that reside within the SGZ of the dentate gyrus region of the hippocampus. NSCs divide asymmetrically creating neural progenitor cells (NPCs) that differentiate and ultimately mature into newborn granule cells [52]. The integration of these newly born neurons into hippocampal circuitry has been observed around three weeks post-birth when they sprout axons that project along the mossy fiber tract to the CA3 region and become capable of receiving excitatory input from the perforant pathway [51,56,57,58]. Beyond the structural support that adult neurogenesis provides to the hippocampus, the functional significance of this mechanism has also been investigated [45,58,59,60,61]. For example, inhibition of adult neurogenesis has been shown to impair hippocampal-dependent behaviors [62,63]. Furthermore, through visualizing immediate early gene expression, such as c-Fos, researchers have been able to investigate neural activation of adult-born neurons during hippocampal-dependent tasks. These studies support the notion that adult neurogenesis contributes to hippocampal recovery and function [64,65,66].

In models of AUDs, inhibition of NSC proliferation and/or adult neurogenesis may contribute to dentate gyrus degeneration more so than cell death [67,68,69,70,71]; see also, [13,47] for review. Indeed, reduced adult neurogenesis has been observed in humans with AUDs and associated with a loss of dentate gyrus granule cells [21,72]. Furthermore, increased neurogenesis is associated with brain recovery in abstinence in male models of alcohol dependence [37,73,74,75,76,77], though not in females or in models that lack dependence [78,79,80,81,82]. Specifically, preclinical studies have found that NSCs and the more fate restricted NPCs reactively proliferated in the SGZ during abstinence from alcohol dependence, an occurrence termed “reactive neurogenesis” [19,73,83,84]. In males, reactive neurogenesis peaks seven days post-binge alcohol exposure and results in a twofold increase in new, mature neurons 28 days later [73,77]. Additionally, several models of brain insult, including traumatic brain injury, ischemia, and seizure, which notably occurs in severe ethanol withdrawal, all exhibit reactive neurogenesis in the hippocampus as well as the subventricular zone [85,86,87,88,89,90,91,92]. However, whether neurons born during alcohol-induced reactive neurogenesis are functionally incorporated into hippocampal circuitry is unknown. Furthermore, understanding whether reactive neurogenesis is a beneficial repair process after brain insult or contributes to aberrant rewiring of hippocampal circuitry, as in the case of seizure, remains a major gap in our knowledge.

Therefore, we examined whether new neurons produced during alcohol-induced reactive neurogenesis are functionally incorporated during a hippocampal-dependent task, the Morris Water Maze (MWM). We used c-Fos immunohistochemistry (IHC) as an indicator of neuronal activity since its expression is increased in granule cells of the dentate gyrus following the MWM [57,93]. We identified these activated, newborn cells by triple-label for bromodeoxyuridine (BrdU) incorporation (birthdate marker on the seventh day of abstinence, the peak of reactive NSC proliferation [73]), c-Fos (neuronal activation) and neuronal nuclei (NeuN; mature neuron). Based on previous work showing that MWM behavior recovers weeks after a four-day binge exposure and a time course consistent with reactive neurogenesis contributing to that recovery [94], we hypothesize that reactive neurogenesis produces neurons capable of integration into hippocampal structure and function.

## 2. Materials and Methods

### 2.1. Subjects

Twenty-one male Sprague-Dawley rats (Charles River Laboratory; ~PND 70) weighing 275–300 g on arrival, were double-housed and allowed to acclimate to the University of Kentucky AAALAC-accredited vivarium for five days before experimental procedures began. All rats were maintained on a regular light cycle (lights on 0600–1800) and allowed ad libitum access to rat chow and water at all times except during the four days of binge alcohol exposure when rat chow was removed at the initial dosing. During the 5-day acclimation period, all rats were individually handled for 3 min per day over 3 consecutive days. All experimental procedures were approved by the University of Kentucky Institutional Animal Care and Use Committee and followed the Guidelines for the Care and Use of Laboratory Animals [95].

### 2.2. Binge Model of An Alcohol Use Disorder

#### 2.2.1. Alcohol Exposure 

**Figure 1:**
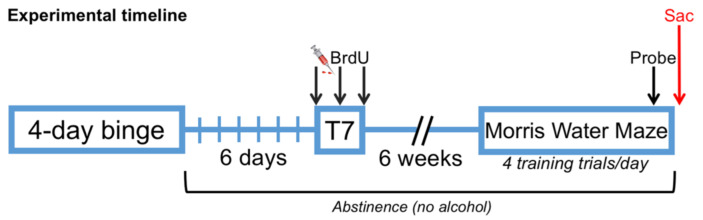
Experimental timeline: Seven days after the final dose of alcohol, all rats were administered 3 i.p. injections of bromodeoxyuridine (BrdU) every 8 h. The rats then underwent 6 additional weeks of undisturbed abstinence in the home cage to allow for maturation of the proliferating cells. Starting on day 49 of abstinence, all animals were trained to perform the Morris Water Maze (MWM) over the course of 4 days. One hour after the final training session on day 52 (the fourth day of training), the platform was removed and all rats were tested in a probe trial. All animals were sacrificed (SAC) 90 min after their final MWM training trial (not including the probe trial).

The experimental timeline is shown in Figure 1. Rats were subjected to a 4-day binge model of an AUD that achieves high blood ethanol concentrations (BECs) similar to those observed in human AUDs [96,97], tolerance, and physical dependence as described previously [73,98,99]. This model produces a well described corticolimbic neurodegeneration and induces reactive, adult neurogenesis [27,83,100,101,102]. Briefly, rats were divided randomly into two groups with similar starting weights and gavaged every eight h for four days with either 25% (*w*/*v*) ethanol in Vanilla Ensure Plus^®^ (*n* = 10; Abbott Laboratories, Columbus, OH) or an isocaloric control diet of Vanilla Ensure Plus^®^ and dextrose (*n* = 11; Fisher). All subsequent doses were titrated based on the animal’s behavioral intoxication score (Table 1). For example, less intoxicated rats (e.g., hypoactive and mildly ataxic) receive more alcohol compared to rats showing stronger behavioral signs of intoxication such as loss of righting reflex. Control rats received the average volume of diet given to the ethanol animals.

#### 2.2.2. Blood Ethanol Concentration (BEC)

Ninety min following the seventh dose of ethanol, tail blood was collected to determine BECs identical to previous studies [98]. Blood samples were centrifuged at 1800× *g* for five min to separate serum and then stored at −20 °C until analysis. All blood serum samples were run in triplicate with frequent calibration to a fresh, 300 mg/dl ethanol standard on an AM1 Alcohol Analyzer (Analox Instruments, Lunenberg, MA, USA).

#### 2.2.3. Withdrawal Observation

Ethanol rats were observed for 30 min of every h for 18 h beginning 10 h after the last dose of ethanol. Withdrawal behavior was scored according to a scale modified from Majchrowicz as described previously (Table 2) [98]. Observed behaviors were recorded with the highest (most severe) numerical score assigned for each hour. These scores were averaged across the 18 h as an index of mean withdrawal severity. Peak withdrawal score was also analyzed for each rat and consisted of the highest withdrawal score observed at any time point across the entire 18 h withdrawal period.

### 2.3. Bromodeoxyuridine Administration

Prior studies have found that peak reactive neurogenesis occurs seven days (T7) after the final dose of alcohol in this model [73,83]. To visualize these newborn neurons, rats were administered bromodeoxyuridine (BrdU; Roche, Mannheim, Germany) to label dividing cells. Beginning exactly 168 h (i.e., T7 days) after the last dose of alcohol, all animals received a total of three BrdU injections (100 mg/kg in 0.9% saline, i.p.), eight hours apart at 7AM, 3 PM, and 11PM [57,103,104]. BrdU injections were administered every eight hours to label a large pool of proliferating cells across T7 that could potentially be recruited for use in the MWM.

### 2.4. Morris Water Maze (MWM)

Studies have shown that 6–8 week old granule cells are preferentially recruited and activated following the MWM task in mice [57]. Therefore, 6 weeks post reactive NSC proliferation (and birth-dating newborn cells with BrdU), we used the MWM task to induce the expression of the immediate early gene, c-Fos, and determined if neurons born during reactive neurogenesis showed c-Fos activation in response to the MWM. The MWM task was conducted similar to previous studies [94,105,106]. Briefly, seven weeks post-binge alcohol exposure when newborn neurons are six weeks old, rats were trained on the MWM task for four days (*n* = 21; control = 11, ethanol = 10). Each day consisted of a block of four 60 s trials where the rat searched for a 13 cm diameter, submerged platform hidden in a 180 cm diameter pool of water dyed black with non-toxic paint. Once the rat located the platform, he was allowed to remain on the platform for 15 s before being placed back in his home cage to begin a four-min inter-trial interval. The maze starting points (N, S, E, W) were the same for all animals in a given day, but each day the sequence of starting points was different (counterbalanced in a Latin square design). For example, day 1 was organized so that the entry points for the four trials were N, S, E, W; entry points on day 2 were W, E, S, N. The platform remained in the same position for all four days. On the final day, approximately one hour after their last trial, the platform was removed and the rats were placed back in the pool at a novel entry point (SW) for a probe trial. The settings were fixed so that if a rat spent 5 s in the target zone (12.7 cm zone around the old platform location), the trial would end. If the rat failed to spend 5 s in the target zone within the first 60 s, the rat was given an additional 60 s to try and complete the task. A video camera and a motion analyzer (EthoVision XT 10, version 10.1, Noldus, Wageningen, The Netherlands) connected to a Dell Precision T3610 computer was used to measure swim path length (cm), latency to the platform or target zone (s), and velocity (cm/s). Daily blocks (four trials) were averaged for each animal and then across treatment groups (control and ethanol diet) to obtain daily means for latency (s), distance (cm), and swim speed (cm/s) ± standard error of the mean (SEM).

### 2.5. Tissue Preparation

Ninety min after their final MWM acquisition trial (52 days following binge alcohol exposure), the rats were administered a lethal dose of sodium pentobarbital (i.p.; Fatal-Plus^®^, Vortech Pharmaceuticals, Dearborn, MI) and transcardially perfused using 0.1 M phosphate buffered saline followed by 4% paraformaldehyde (PFA). Previous studies utilize the 90 min time point for visualization of c-Fos activity in hippocampal neurons [65,107]. Brains were removed and post-fixed for 24 h in 4% PFA, then stored in 0.1M phosphate buffered saline pH 7.4 (PBS, Gibco, Life Technologies, Grand Island, NY, USA) until sectioning. Twelve series of coronal sections were cut at 40 µm on a vibrating microtome (Leica VT1000S, Wetzlar, Germany) starting randomly mid striatum through approximately Bregma −8.52. Sections were stored in series, in 24-well plates with cryoprotectant at −20 °C until immunohistochemical processing.

### 2.6. Immunohistochemistry

A subset of ethanol and control rats (*n* = 3/group) were selected for exhaustive quantification of triple-label immunohistochemistry (IHC) across the dorsal dentate gyrus. To minimize non-alcohol induced differences, the rats were chosen based on similar pre-binge weights. For the ethanol-treated rats, care was taken to ensure that intoxication and withdrawal scores were close to average values. Triple fluorescent IHC for BrdU (newly born cell), c-Fos (MWM-activated cell), and NeuN (mature neuron) was conducted similar to the protocol described in Geibig [93]. Every 12th free-floating section was rinsed in 0.1M PBS + 1% Triton^®^X-100 (PBST; Acros Organics, NJ, USA). Tissue was then incubated for 30 min in 1 N HCl at 37 °C and neutralized with 0.1 M Boric acid pH 8.5. Following PBST rinses, sections were then incubated in PBST with 15% normal goat serum (Vector Laboratories, Burlingame, CA, USA) containing antibodies for c-Fos (1:200, SC7202, Santa Cruz Biotechnology Inc., Dallas, TX, USA), BrdU (1:400, H7786, Accurate Chemical and Scientific Co., Westbury, NY, USA), and NeuN (1:10,000, MAB377, Millipore, Temecula, CA, USA) for three h at room temperature followed by 42 h at 4 °C. Next, sections were rinsed with PBST and incubated in PBST with 15% normal goat serum and Alexa Fluors^®^ goat anti-rabbit IgG 488, goat anti-rat IgG 546, and goat anti-mouse IgG 633 secondary antibodies (all 1:200, Invitrogen^™^ Molecular Probes^®^, Eugene, OR) for 90 min in a light-proof box at room temperature. Tissue was then rinsed in PBST, mounted onto glass slides, allowed to dry, coverslipped with ProLong^®^ Gold antifade reagent (Molecular Probes^®^ by Life Technologies, Eugene, OR, USA), and left to cure for two nights in the dark.

### 2.7. Quantification

All fluorescently-labeled cells were quantified exhaustively on a single hemisphere (480 µm between sections), between Bregma −2.28 and −5.52 mm as determined by Paxinos and Watson, 2009 [108]. Cells were quantified using a Leica TCS SP5 inverted laser scanning confocal microscope (Wetzlar, Germany). Z-plane optical stacks were collected at 1 µm thickness using a 20× objective. Multiple z-stacks were taken across each section to capture the entire granule cell layer (GCL; 2–5 z-stacks per section, 6–8 sections per brain). Immediately, after each z-stack collection, the images were visually inspected to determine if any triple labeling occurred. This was accomplished using the Leica parent software by hiding the NeuN (blue) channel and looking for overlap of BrdU+ (red) and c-Fos+ (green) cells (i.e., overlapped cells will appear yellow). If it was suspected that a cell may be triple-labeled, then the cell of interest underwent an additional z-stack at a higher magnification (63× lens).

All Z-stack images were viewed and manually quantified using Image-Pro Plus software (version 3.6 windows, Image-Pro Plus, Media Cybernetics, Rockville, MD). This software allowed viewing of all three channels (cell marker images) and the merged images simultaneously. For each z-stack, the series of images were compiled such that each channel and the merged image could be visualized and used to count all BrdU+ and c-Fos+ cells in the GCL. This 100% sample fraction approach was chosen over stereology as BrdU+ cells and c-Fos+ cells are fewer than 100 and heterogeneously scattered across the GCL [109]. Furthermore, difficulty in determining section thickness, i.e., visualizing accurately upper and lower boundaries of the tissue due to the lack of background independent of immunoreactivity in the fluorescent signal, ruled out the use of true stereological approaches [109]. Due to the fluorescent preparation and the extensive number of NeuN+ cells, NeuN was only used to confirm that the cell was a mature neuron. All BrdU+ and c-Fos+ cells were counted exhaustively across the GCL and then each cell was determined to be either BrdU+/NeuN+, BrdU only, c-Fos+/NeuN+, c-Fos only, or c-Fos+/BrdU+/NeuN+ [93]. Cell counts were then totaled for each animal (2–5 z-stacks per brain section, 6–8 sections per animal) and means for control and ethanol animals were calculated ± SEM.

### 2.8. Statistical Approaches

All data were analyzed with GraphPad Prism (version 7, GraphPad Software, La Jolla, CA, USA) and reported as mean ± SEM. Histological data were analyzed by student’s t-test. MWM data were analyzed by two-way repeated measures ANOVA (diet x day) and followed by Bonferroni’s multiple comparisons test when appropriate. MWM probe trial data were analyzed by student’s t-test. P-values were accepted as significant when *p* < 0.05.

## 3. Results

### 3.1. AUD Model Data

Ethanol rats had a mean intoxication score of 1.8 ± 0.1 (ataxia, with elevated abdomen; Table 1 and Table 3). The ethanol dose averaged 9.7 ± 0.2 g/kg/d which resulted in mean BECs of 401 ± 14 mg/dl, as measured on the third day of the binge. Mean withdrawal scores were 1.4 ± 0.2 (tail tremors) and peak withdrawal scores were 3.6 ± 0.1 (chattering teeth) on the behavioral withdrawal scale (Table 2 and Table 3). With the exception of BECs being slightly higher than usual, these values are similar to previous reports [98]. Importantly the subgroup of rats used for IHC analyses were no different than the entire group as used for the MWM (Table 3).

### 3.2. Abstinence from Alcohol Treatment Improves Acquisition of the MWM Task

**Figure 2:**
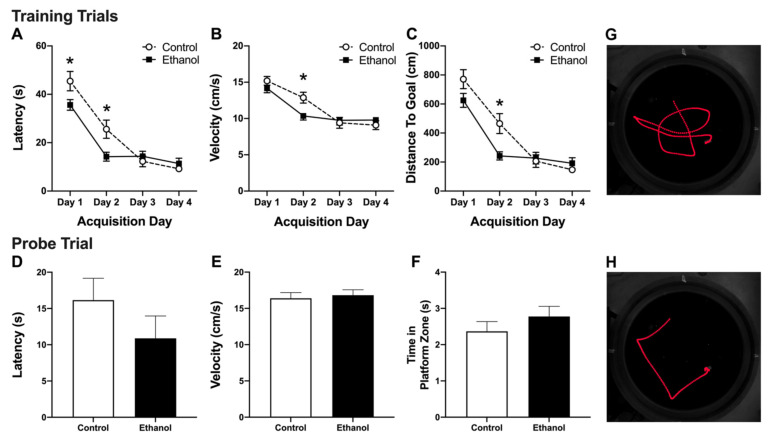
Morris water maze (MWM) performance: Two-way repeated measures analysis of the 4-day MWM training revealed a significant main effect of acquisition day and an acquisition day by alcohol experience interaction on (**A**) latency (**B**) swim velocity, and (**C**) distance travelled, but no main effect of prior alcohol exposure alone. Planned comparisons revealed significantly better MWM performance as assessed by latency, velocity, and distance travelled in the alcohol-treated group (*n* = 10) compared to controls (*n* = 11) on training days 1 (latency) and 2 (latency, velocity, and distance travelled). Symbols represent means for each day. On the fourth day following the MWM training trials, all animals underwent a probe test in which the platform was removed. Unpaired t-tests revealed no significant differences between the alcohol-treated animals and controls in the (**D**) latency to the platform area, (**E**) swim velocity, and (**F**) time spent in the previously learned platform zone. Representative MWM traces from a (**G**) control and (**H**) ethanol-treated rat on day 2 of acquisition. Bars represent means ± standard error of the mean (SEM). * *p* < 0.05.

Conducted identical to Nickell, Thompson et al., 2020 [94], the MWM task was used to induce c-Fos activation in dentate gyrus granule cells [93,103]. Two-way repeated measures ANOVAs of the four day acquisition period revealed a significant main effect of day for latency (Figure 2A, F_(3,57)_ = 64.2, *p* < 0.0001), swim velocity (Figure 2B, F_(3,57)_ = 42.41, *p* < 0.0001), and distance traveled to the platform (Figure 2C, F_(3,57)_ = 63.8, *p* < 0.0001) as rats, regardless of group, decreased their time, swim speed, and distance to find the platform. Additionally, there was a significant diet x day interaction for all measurements (Latency: F_(3,57)_ = 4.70, *p* = 0.0053; Velocity: F_(3,57)_ = 3.818, *p* = 0.0148; Distance traveled: F_(3,57)_ = 4.71, *p* = 0.0053). Bonferroni post-hoc comparisons revealed that ethanol-treated rats exhibited decreased latency to the platform on day 1 and 2 (*p* <0.05, Figure 2A) and a reduction in swim velocity and (*p* < 0.05, Figure 2B) and in the distance traveled to the platform on day 2 (*p* < 0.05, Figure 2C) compared to controls. Overall, these results suggest that animals exposed to alcohol 7 weeks earlier acquire the MWM task quicker than rats fed a control diet. Furthermore, this improvement in locating the platform is not a result of increased swim speed (velocity), as the ethanol-treated rats appear to swim slower than the controls, but was a result of a more efficient swim trajectory to the platform.

### 3.3. Alcohol Treatment Has No Effect on Recall of the MWM

On day four of MWM training, one hour after the final acquisition trial, rats were placed back in the MWM apparatus for a probe trial. During the probe trial, the platform was removed and the rats were tested on their ability to locate the prior platform location, or the platform zone. Despite the differences in acquisition of the MWM task, unpaired *t*-tests did not reveal any differences in latency to the platform zone (Figure 2D; t(19) = 1.218, *p* = 0.2379), swim velocity (Figure 2E; t(19) = 0.3975, *p* = 0.6954), or time in the platform zone (Figure 2F; t(19) = 1.051, *p* = 0.3066), between the ethanol and control groups on the probe trial.

### 3.4. Abstinence from Ethanol Increases Neurogenesis in the Dentate Gyrus (DG)

**Figure 3:**
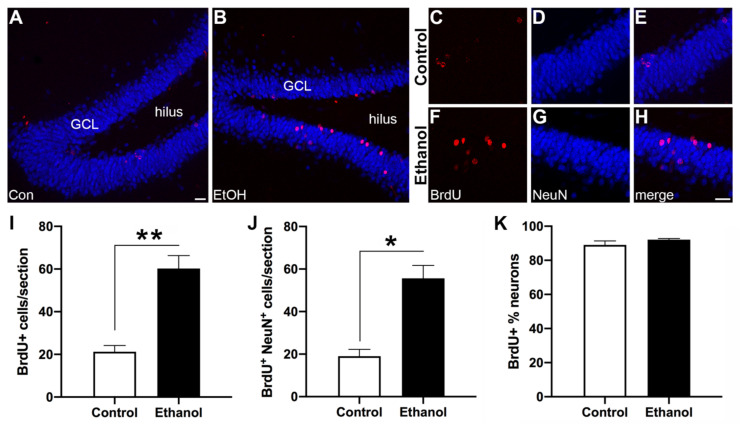
Reactive neurogenesis: Representative images of the dentate gyrus from control (**A**) and ethanol-exposed (**B**) animals at T7 highlighting the distribution of bromodeoxyuridine (BrdU)/Neuronal Nuclei (NeuN) immunoreactivity. Higher magnification images for individual fluorochromes illustrating BrdU+ alone (red), NeuN+ alone (blue), and merged for both a control (**C**–**E**) and an ethanol-treated (**F**–**H**) rat. (**I**) Unpaired t-tests revealed significantly more BrdU+ cells and (**J**) more BrdU+/NeuN+ co-labelled neurons in the ethanol-treated animals compared to the controls. (**K**) Of the BrdU+ cells only, there is no significant difference between the ethanol and control groups in the percent of BrdU+/NeuN+ co-labelled neurons. GCL—granule cell layer. Bars represent means ± SEM. * *p* < 0.05, ** *p* < 0.01. Scale bars = 40 µm.

As shown in Figure 3, BrdU-labeled cells are scattered around the inside half of the dentate gyrus GCL with granule cells expressing NeuN as expected. Six weeks after reactive NSC proliferation (seven weeks after their final dose of alcohol), ethanol-treated rats showed an increase in the number of BrdU+ cells compared to controls (Figure 3I; t_(4)_ = 5.796; *p* = 0.0044). Additionally, ethanol animals also exhibited an increase in the number of BrdU+/NeuN+ cells compared to controls (Figure 3J; t_(4)_ = 5.347; *p* = 0.0059), which confirms reactive neurogenesis occurred in the ethanol group. Despite these increases in newly generated neurons in the ethanol-exposed rats, there was no difference in the proportion of BrdU+/NeuN+ cells within the whole BrdU+ cellular population in the ethanol-treated animals compared to the controls (Figure 3K; t_(4)_ = 1.275; *p* = 0.2713).

### 3.5. Prior Ethanol Exposure Has No Effect on MWM-Induced c-Fos Expression

**Figure 4:**
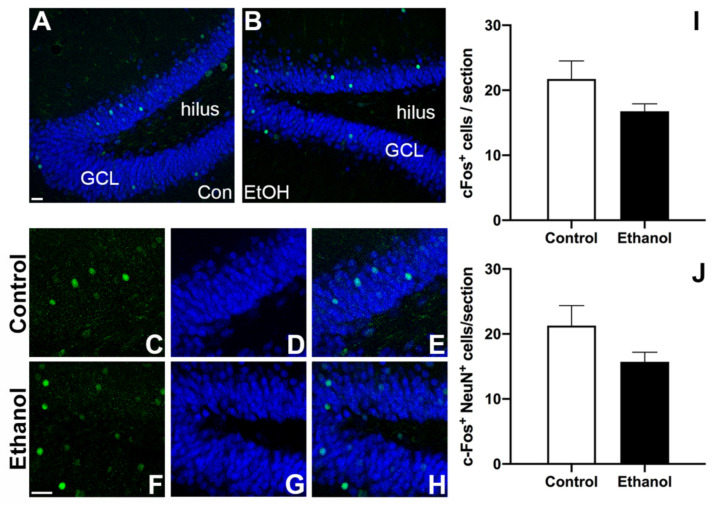
Neuronal activation of the Morris Water Maze (MWM) task: (**A**,**B**) Representative images of the dentate gyrus from control (**A**) and ethanol-exposed (**B**) animals highlighting the distribution of c-Fos/NeuN immunoreactivity. Higher magnification images for individual fluorochromes illustrating c-Fos+ alone (green), NeuN+ alone (blue), and merged for both a control (**C**–**E**) and an ethanol-treated (**F**–**H**) rat. Unpaired *t*-tests revealed no significant differences between the control and ethanol-exposed animals in (**I**) the number of cFos+ cells and (**J**) the number of cFos+/NeuN+ co-labelled neurons. GCL—granule cell layer. Bars represent means ± SEM. Scale bars = 40 µm.

Next, we investigated the effect of prior binge ethanol exposure and subsequent abstinence on hippocampal neuronal activation. As shown in Figure 4, c-Fos+ cells were scattered throughout the entire GCL and c-Fos/NeuN+ expression was apparent in the GCL of both groups. Unpaired t-tests revealed no significant differences in the number of c-Fos+ cells (Figure 4I; t_(4)_ = 1.656; *p* = 0.1731) or the amount of c-Fos+ neurons (c-Fos+/NeuN+ co-label) (Figure 4J; t_(4)_ = 1.624; *p* = 0.1797). Taken together, these data suggest that ethanol consumption did not alter hippocampal neuronal activation in response to the MWM task.

### 3.6. Ethanol Does Not Alter MWM-Induced Activation of Adult Born Neurons

**Figure 5:**
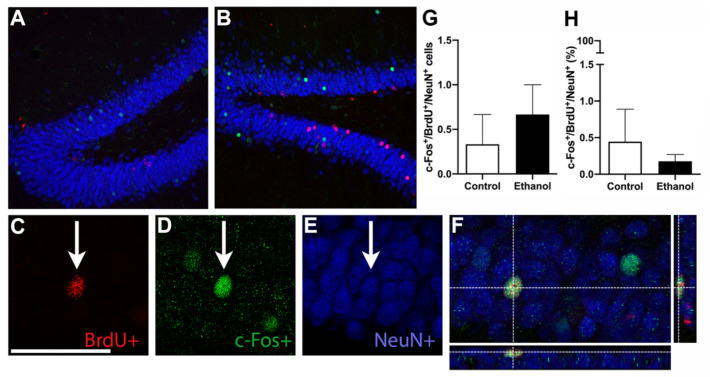
Incorporation of newly generated neurons: (**A**,**B**) Representative images of the dentate gyrus from control (**A**) and ethanol-exposed (**B**) animals for bromodeoxyuridine (BrdU)/c-Fos/NeuN triple-label immunoreactivity. (**C**–**F**) Higher magnification images for individual fluorochromes illustrating a BrdU+ cell (**C**, red), a c-Fos+ cell (**D**, green), and a NeuN+ cell (**E**, blue), as well as a merged and rendered orthogonal z-stack of the triple-labelled (BrdU+/c-Fos+/NeuN+) cell (**F**). Unpaired *t*-tests revealed no significant differences in the (**G**) number or (**H**) percent of BrdU+ cells that also expressed cFos and NeuN (triple-labelled) between the controls and ethanol-exposed animals. GCL—granule cell layer. Bars represent means ± SEM. Scale bars = 40 μm.

Ultimately, we were interested in examining whether there were changes in MWM-induced neuronal activation (c-Fos+) in the population of neurons born during the alcohol-induced reactive neurogenesis on day 7 of abstinence (BrdU+/c-Fos+/NeuN+). BrdU+ expression was evaluated throughout the GCL, with each BrdU+ cell further examined for c-Fos+ and NeuN+ expression (triple-label) as shown in representative images in Figure 5. Unpaired t-tests revealed no significant difference in the number of triple-labeled cells (BrdU+/c-Fos+/NeuN+) between ethanol-exposed and control rats (Figure 5G; t_(4)_ = 0.7071; *p* = 0.5185). Additionally, the proportion of BrdU+ cells that also expressed c-Fos and NeuN (i.e., cells that were triple-labeled) was not different between ethanol and control groups (Figure 5H; t_(4)_ = 0.591; *p* = 0.5861). Taken together, this suggests that ethanol treatment does not alter the recruitment of adult born neurons into functional hippocampal-dependent neuronal ensembles.

## 4. Discussion

Adult neurogenesis is essential to hippocampal integrity with adult born neurons playing roles in hippocampal-dependent functions such as learning and memory [45,57,58,59,60,61,62,63,64,65,66,93]. In multiple models of brain insult, reactive adult neurogenesis may contribute to repair and recovery of the hippocampus [19,37,84,87,88,89,90,91,92,94,110]. The goal of this project was to determine for the first time, if neurons generated during reactive neurogenesis were capable of activation in response to the MWM, which was achieved in spite of a few unexpected findings. Collectively, these data suggest that new neurons born during alcohol-induced reactive neurogenesis are incorporated into hippocampal function at the same rate as under control conditions, as indicated by similar MWM-induced c-Fos expression in BrdU+ newborn neurons. Only newborn cells that were labeled with BrdU during reactive neurogenesis, survived six weeks, and expressed a mature neuronal marker (NeuN), and were activated in response to a hippocampal-dependent task (c-Fos; MWM) become triple-labeled, which suggested that these newborn neurons were integrated into the hippocampal circuitry necessary for learning and memory performance [57,93]. The number of triple-labeled cells was similar for both groups supporting that a) neurons born during reactive neurogenesis following alcohol dependence can be activated in response to the MWM task and are potentially functional and b) recruitment of new cells into hippocampal circuitry occurs at a similar rate. Following alcohol dependence and damage, reactive neurogenesis resulted in approximately a threefold increase in new neurons indicated by BrdU+/NeuN+ cell counts seven weeks-post binge, confirming previous reports [73,83,94]. More than 90% of these BrdU+ cells co-expressed NeuN indicating that the majority of cells labeled on T7 that survived to T52 became mature neurons. This result is consistent with previous studies that show increases in hippocampal neurogenesis during abstinence from alcohol dependence [73,74,75,78]. In addition, the number of c-Fos+ cells in either group was similar to that reported previously for rats with similar methods [93,110]. Triple-labeled cells were observed in both ethanol and control animals, which indicated that the neurons generated during reactive neurogenesis were capable of activation in response to the MWM. That c-Fos was activated as a direct result of the MWM is supported by previous research where rats not exposed to MWM express no detectable levels of c-Fos [103]. To confirm that c-Fos expression is not detectable in animals not exposed to the water maze in our hands, tissue sections from multiple animals from a similar four-day binge study were stained for c-Fos alongside a positive control (MWM rats). No c-Fos+ cells were detectible in animals that did not undergo the MWM (data not shown).

Despite the increase in neurogenesis observed in ethanol rats, the number of cells activated in response to the MWM (measured with c-Fos; Figure 4) did not differ between ethanol and control rats. This result is in agreement with neuronal ensemble studies, which find that only a small proportion of neurons in any region, especially in the dentate gyrus where minimal neuronal activation is observed, are necessary for behavioral expression and/or memory encoding [111,112,113,114,115]. Furthermore, we observed no preferential recruitment of newborn neurons into the MWM-induced hippocampal ensemble, but instead were incorporated at the same rate as in controls. These data may suggest that a particular number of newborn neurons are sufficient for learning and memory processes, especially considering that too many newborn cells may be problematic for function [71,86]. The results from the current study were consistent with past work [93], where reactive neurogenesis post-stroke in mice resulted in an equal number of MWM-activated young neurons (BrdU+/c-Fos+/NeuN+) compared to controls. While we did not observe increased activation of newborn neurons in response to the MWM task, others [57] found that six-week old neurons are preferentially recruited into a MWM neuronal ensemble compared to older granule cells. However, other groups have observed that c-Fos expression is similar in both newborn neurons and older granule cells following MWM performance in rats [103]. One potential reason for this difference can be attributed to the temporally limiting properties of the BrdU labeling window utilized. While peak reactive neurogenesis occurs on the seventh day of abstinence, neurogenesis following alcohol dependence is elevated over a number of days (T5–7) [73,83]. As BrdU only labels cells that are actively dividing at the point of injection, it is possible that by only administering BrdU at T7, we are limiting our ability to visualize the full scope of neurons generated during this reactive period [116]. By using a longer time course of BrdU administration or viral birthdating methods, future studies would be able to observe a more complete picture of the progenitor population following alcohol dependence. On the other hand, this observed difference in newborn neuronal activation may be the result of the very low number of MWM-activated neurons (c-Fos+ neurons), similar to that reported by Geibig et al., 2012 [82] though lower than Kee et al., 2007 [55]. The most significant changes in c-Fos occur during the initial session in a multiple session training protocol or during recall of a previously learned task (for review see [117]). Kee et al., 2007 [55] examined c-Fos expression in neurons activated during recall of a previously learned behavior, i.e., the probe trial of the MWM task, while the current study was timed to performance of the last trial. As such, future studies could examine the activation of newborn neurons during initial learning sessions, especially at points where there are performance differences between groups, or following the probe trial. However, the lack of group differences observed during performance of the probe trial in the current experiment suggests that newborn neuron activation would likely be similar between alcohol and control groups at this late abstinence time point.

Neurons generated during alcohol-induced reactive neurogenesis may differ from those generated under control conditions. Under normal conditions, newborn cells may originate from various stem or progenitor cells, whereas cells born during reactive neurogenesis result from the differential activation of type 1 NSC and NPCs [83]. Ectopic neuroblasts have also been observed in adolescent models of an AUD, though this has not been reported in adult rats to date [73,118]. Furthermore, recent studies also suggest that neurons born as a result of brain insult or injury are utilized specifically for the repair of damaged circuits (review [90,119]). In the current study, the majority of these newly generated neurons were not incorporated into the MWM-induced ensemble. As only a limited number of neurons are in any neuronal ensemble and required for behavioral expression, therefore, it is possible that those neuroblasts generated during alcohol-induced reactive neurogenesis may be destined to repair disrupted circuits and not necessarily for integrating into new ensembles. Alternatively, these newborn neurons may contribute to additional sequelae of alcohol dependence such as later development of seizures or epilepsy for which there is an increased risk in AUDs [120,121].

Whether reactive neurogenesis is globally beneficial is still up for debate [37,85,90,91,92,122]. While the impact and extent of reactive neurogenesis likely depends on the disease model and perhaps the type of increased neurogenesis, in models of AUD specifically, the majority of studies lean towards it contributing to hippocampal recovery. Findings from multiple groups observe beneficial effects of increased neurogenesis during abstinence from alcohol dependence such as recovery of dentate gyrus granule cell numbers and hippocampal-dependent functions [73,74,83,84,94]. Specifically, our recent work showed that MWM performance returns to control levels 35 days post binge exposure concurrent with normalization of dentate gyrus granule cell number in male rats [83,94]. In the current study, ethanol-treated rats not only recovered but surprisingly performed better on the MWM task (i.e., quicker latency to locate the platform) compared to controls (Figure 2). Thus, this correlation between increased neurogenesis and enhanced learning performance supports that reactive neurogenesis is beneficial, however there are caveats. (1) As discussed in our prior work [29], reactive neurogenesis via stem cell activation has potential consequences of depleting the stem cell pool, an effect which has long term implications for the structure and function of the hippocampus. (2) The specific role of reactively born neurons is not clear. Blunting reactive neurogenesis to control levels with the DNA alkylating agent, temozolomide, did not similarly blunt behavioral recovery [94]. The limited extent of knock down may explain this lack of effect and studies are ongoing with better, transgenic tools [123].

It is of note that these prior studies have only used adult, male rats. Similar reactive increases in hippocampal neurogenesis during abstinence from alcohol are exhibited in adult females, but the dentate gyrus granule cell recovery was not observed [78,82,118]. The functional significance of this process in female rats is under investigation. Furthermore, adolescent rats show ectopic, aberrant neurogenesis, which may contribute to ongoing pathology [77] while in non-dependent models of adolescent alcohol exposure, adult neurogenesis is inhibited long-term [76,80,81]. Recent work in female mice found that chronic alcohol exposure resulted in aberrant integration of newborn neurons and impairment on novelty recognition tasks [71]. Obviously, differences in these two studies—rodent models, exposure methods, and learning tasks—complicate our understanding of how these new cells contribute to hippocampal recovery and function. As increased neurogenesis correlates with improved MWM performance, and alcohol dependence increases neurogenesis in abstinence, reactive neurogenesis is strongly implicated in this improvement [58,93,124]. Yu et al. (2019) discovered that the improved performance on the MWM task in rats with intact neurogenesis was due to the development of a more efficient behavioral strategy [125]. This could explain the improved task performance in our ethanol-treated rats, despite slower swim speeds. Furthermore, despite these positive associations, many questions remain unanswered regarding the role and function of reactively born neurons following alcohol dependence.

Interestingly, this improvement in learning did not result in elevated neuronal activation. There are several possibilities related to both neurogenesis and approaches to studying neuronal ensembles and integration that may underlie this lack of difference. First, the ratio of activated newborn neurons to the number of newborn neurons differs between groups. The dentate gyrus may only be able to integrate a set number of newborn cells [103]. Reactive neurogenesis following alcohol dependence produces a large number of new progenitors (e.g., 4–5-fold increase in NPC proliferation), but this only results in a twofold increase in the number of new neurons suggesting decreased survival of newborn cells [73]. Second, MWM-induced activation of newborn neurons could be stronger in the ventral rather than the dorsal dentate gyrus [64], although this seems counterintuitive to what is known about the role of the dorsal hippocampus in the MWM [126,127]. As we focused on the dorsal dentate gyrus in the current experiment, it is possible that increased neuronal activation of newborn neurons may have occurred in the ventral dentate gyrus. A final potential reason for the divergence between behavioral performance and the neuronal activation we observed may be a result of the neuronal activation marker used. Future studies could investigate additional immediate early genes, such as zif268 or Arc in place of c-Fos as each has a slightly different expression profile, while still being indicative of activation in response to a hippocampal-dependent task [128,129]. For example, c-Fos as a marker of hippocampal activation may underrepresent the full extent of neuronal activation. On the other hand, Zif268 exhibited lower basal expression in the dentate gyrus, however when activated, expression is roughly four times higher than that of c-Fos [93]. Therefore, utilizing a different immediate early gene may uncover a larger population of activated neurons. However, despite this lack of increased behaviorally-induced newborn neuron activation in our ethanol-treated animals, the current study does provide new evidence that these reactively-born newborn neurons can be functionally incorporated into new hippocampal-dependent neuronal ensembles.

## 5. Conclusions

In conclusion, these data support the hypothesis that neurons generated during alcohol-induced reactive neurogenesis are capable of becoming incorporated into hippocampal networks. The number of triple-labeled cells, indicating task-activated newborn neurons, did not differ between ethanol and control animals which suggests that activation is similar between cells generated during basal neurogenesis and during reactive neurogenesis. Although the number of activated neurons is very small, only a small number of new neurons may contribute to the hippocampal network for a given task [93,130,131]. In addition, activation in response to MWM represents only one facet of functional integration [93]. Many questions remain as we acknowledge that a triple-labeled cell may not necessarily be functionally normal. Electrophysiological studies of these reactively born cells are necessary to investigate connectivity and electrophysiological properties to determine whether neurons born during reactive neurogenesis undergo normal functional integration. In clinical studies, abstinence from alcohol has led to improvements in not only brain volume, but hippocampal-related learning and memory tasks as well [36,38,42,132]. While the mechanism of brain recovery in humans suffering from AUDs is not known, considering the role of reactive, adult neurogenesis and its contribution to hippocampal integrity remains an enticing explanation. Further understanding of the functional significance of reactive neurogenesis can provide insight into hippocampal recovery and perhaps the development of more targeted and effective treatments for AUDs.

## Figures and Tables

**Table 1:** Intoxication behavior scale and dosing.

Score	Intoxication Behavior	Dose
0	Normal	5 g/kg
1	Hypoactive, mild ataxia	4 g/kg
2	Ataxia (abdomen elevated)	3 g/kg
3	Delayed righting reflex Severe ataxia (abdomen drags)	2 g/kg
4	Loss of righting reflex	1 g/kg
5	Loss of eyeblink reflex	0 g/kg

**Table 2:** Withdrawal Severity Scale.

Score	Withdrawal Behaviors
1.0	hyperactivity
1.4	tail tremor
1.6	tail spasm
2.0	caudal tremor
2.4	splayed limbs
2.6	general tremor
3.0	head tremor
3.4	wet dog shake
3.6	chattering teeth
3.8	spontaneous convulsion

**Table 3:** Binge parameters for immunohistochemistry (IHC) versus MWM cohorts.

Group	Subjects	Intox Score	Dose (g/kg/day)	BEC (mg/dl)	Mean WD	Peak WD
MWM	EtOH = 10; Con = 11	1.8 ± 0.1	9.7 ± 0.2	401 ± 14	1.4 ± 0.2	3.6 ± 0.1
IHC	EtOH = 3; Con = 3	1.8 ± 0.1	9.8 ± 0.3	400 ± 10	1.0 ± 0.2	3.7 ± 0.1

Intox—intoxication; BEC—Blood Ethanol Concentration; WD—withdrawal; MWM—Morris Water Maze; EtOH—ethanol group; Con—control group; IHC—immunohistochemistry.

## Data Availability

The datasets generated for this study are included in the article. Further inquiries can be directed to the corresponding author.
